# Characterisation of DOG-1 Expression in Salivary Gland Tumours and Comparison with Myoepithelial Markers

**DOI:** 10.1007/s12105-018-0917-3

**Published:** 2018-04-18

**Authors:** Syed A. Khurram, Paul M. Speight

**Affiliations:** Unit of Oral and Maxillofacial Pathology, School of Clinical Dentistry, 19 Claremont Crescent, Sheffield, S10 2TA UK

**Keywords:** DOG-1, Salivary gland tumours, Acinic cell carcinoma, Secretory carcinoma, Myoepithelial, Luminal

## Abstract

DOG1 is an established diagnostic marker for gastrointestinal stromal tumours (GIST), but has been reported in salivary gland tumours (SGT) as an acinar and intercalated duct marker. However, its specificity and distribution is not well established. The aim of this study was to evaluate the diagnostic utility of DOG-1 expression in SGT in addition to comparing it with myoepithelial markers. Normal salivary tissue and SGT (n = 184) were examined for expression of DOG1 and a range of myoepithelial markers. SGT included: acinic cell carcinoma (ACC, n = 15), secretory carcinoma (SC, n = 9), pleomorphic adenoma (PA, n = 49), carcinoma ex-PA (Ca ex-PA, n = 11), adenoid cystic carcinoma (AdCC, n = 20), polymorphous adenocarcinoma (PAC, n = 6), myoepithelioma (n = 6), myoepithelial carcinoma (MC, n = 2), basal cell adenoma (BCA, n = 14), canalicular adenoma (CA, n = 19), mucoepidermoid carcinoma (MEC, n = 11), oncocytoma (n = 2), adenocarcinoma NOS (AdNOS, n = 4), basal cell adenocarcinoma (BCAC, n = 2), salivary duct carcinoma (SDC, n = 3) and papillary cystadenocarcinoma (PCAC, n = 1). Normal acini and ACC (14/15) showed strong luminal DOG1 staining; SC were largely negative with only focal expression in 3/9 cases. Luminal staining was seen in PA (14/49), PAC (4/6), Ca ex-PA (4/11) and AdCC (6/20). 8/11 MEC showed luminal and/or mucous cell staining. No staining was seen in myoepithelioma, MC, CA, adNOS and BCAC. BCA showed strong staining of myoepithelial cells in some cases (5/14). Variable myoepithelial DOG1 staining was seen in PA, Ca ex PA, BCA, SDC and PCAC which was not as consistent as myoepithelial markers such as calponin, p63 and αSMA. Absence of DOG1 can differentiate ACC from SC, but staining is variable in PA, PLGA and Ca ex-PA. Myoepithelial staining in some tumours but not in normal gland suggests a wider distribution in SGT than originally envisaged.

## Introduction

Diagnosing salivary gland tumours can be challenging due to the heterogeneity of the cellular differentiation, morphogenesis and histological patterns. Many different tumour entities share similar histological patterns, which further complicates diagnosis.

In 2004, ‘Discovered on GIST-1’ (DOG1) was shown to be highly expressed in a high proportion of gastrointestinal stromal tumours (GISTs) [1–3]. Subsequently, a number of in vivo studies revealed DOG1 to be a calcium activated chloride channel expressed on secretory epithelium in mouse models [4, 5]. More recently, DOG1 expression has been reported in salivary gland tumours [6–8], in particular, as a marker for acinic and intercalated duct cells. Some studies have suggested that the DOG1 protein may be essential for salivary gland secretion with a possible role in salivary gland tumourigenesis [5, 9]. However, its pattern of expression and specificity in a range of tumours has not been fully established. Strong staining is seen at the luminal aspect of acinar cells in normal glands, and luminal staining has been shown in the acini in ACC [10], and in small ductal structures in PAC and epithelial myoepithelial carcinoma (EMC) [6]. Luminal and abluminal staining has been described in BCA and AdCC [6, 11]. Our clinical experience has shown expression by myoepithelial cells in some instances, a finding not reported to date.

The aim of this study was to study the expression pattern, specificity and diagnostic potential of DOG-1 in salivary gland tumours.

## Methods

Normal parotid and submandibular gland tissue and SGT (n = 184) were examined for expression of DOG1 and a range of myoepithelial and cytokeratin markers using immunohistochemistry (IHC) on cases retrieved from the department archive. These included acinic cell carcinoma (ACC, n = 15), secretory carcinoma (SC, n = 9), pleomorphic adenoma (PA, n = 49), carcinoma ex-PA (Ca ex-PA, n = 11), adenoid cystic carcinoma (AdCC, n = 20), polymorphous adenocarcinoma (PAC, n = 6), myoepithelioma (n = 6), myoepithelial carcinoma (MC, n = 2), basal cell adenoma (BCA, n = 14), canalicular adenoma (CA, n = 19), mucoepidermoid carcinoma (MEC, n = 11), oncocytoma (n = 2), adenocarcinoma NOS (AdNOS, n = 4), basal cell adenocarcinoma (BCAC, n = 2), salivary duct carcinoma (SDC, n = 3) and a papillary cystadenocarcinoma (PCAC, n = 1).

The diagnosis of the cases was confirmed by H&E staining and examination under the light microscope. Tumours were classified according to WHO 2017 guidelines [12] and current literature. FISH analysis for the *ETV6* rearrangement was used as a gold standard for the diagnosis of all included SC.

### Immunohistochemistry

IHC for DOG1 was performed on the entire cohort. For comparison a range of other ‘myoepithelial markers’ were also studied in a proportion of tumours including S100, αSMA, p63, calponin and CK14 as previously described [8]. Multiple pilot assays were undertaken to determine the optimum dilution and conditions (Table 1).

**Table 1:** Details of primary antibodies used in the study

Antibody	Clonality	Dilution	Retrieval	Supplier
DOG 1	Mouse monoclonal	1:250	EDTA	Dako®, Cambridgeshire, UK
S100	Rabbit polyclonal	1:2,000	EDTA	Dako®, Cambridgeshire, UK
SMA	Mouse monoclonal	1:75	Citrate	Dako®, Cambridgeshire, UK
P63	Mouse monoclonal	1:25	EDTA	Dako®, Cambridgeshire, UK
CK 14	Mouse monoclonal	1:20	Citrate	Abcam®, Cambridge, UK
Calponin	Rabbit monoclonal	1:100	Citrate	EP798Y, Abcam®, Cambridge, UK

4 µ thick sections from formalin-fixed, paraffin embedded tissue blocks were used for IHC staining. Sections were deparaffinised in xylene followed by incubation in ethanol for 5 min each. Endogenous peroxidase was blocked by incubation in 3% methanolic H_2_O_2_ blocking solution for 20 min followed by a wash in phosphate buffered saline (PBS). Antigen retrieval was carried out by placing the slides in a heat-resistant plastic container filled with citrate or EDTA buffer solution in a microwave for 10 min on high power. The slides were left to cool for 2 min, placed in PBS to avoid dehydration and blocked with 100% normal serum for 30 min at room temperature (RT). Serum was removed followed by addition of the primary antibody overnight at 4 °C in a humidified chamber. Omission of primary antibody served as the negative control.

After overnight incubation, unbound primary antibody was removed and the slides washed twice for 5 min with PBS. The secondary antibody and ABC solution were prepared according to the manufacturer’s instruction (Vectastain Elite kits, Vector Laboratories, Burlingame USA). Sections were covered with secondary antibody for 30 min followed by two washes in PBS and addition of avidin biotin complex (ABC) solution for 30 min at RT. After two further washes in PBS 3,3′-diaminobenzidine (DAB, Vector laboratories) was applied to the sections for 5–8 min and the colouring reaction stopped using distilled water. Sections were counterstained with Mayer’s haematoxylin, mounted in DPX mounting media and left to dry at RT.

IHC staining was assessed subjectively under standard light microscopy, taking into account the pattern and localisation of the staining. Stained sections were photographed using a digital imaging system (cell^D) and a digital camera attached to a light microscope (Olympus, UK). As previously described, myoepithelial staining was considered positive in the correct morphologic context to ensure exclusion of stromal cells [8].

## Results

### DOG-1

**Fig. 1:**
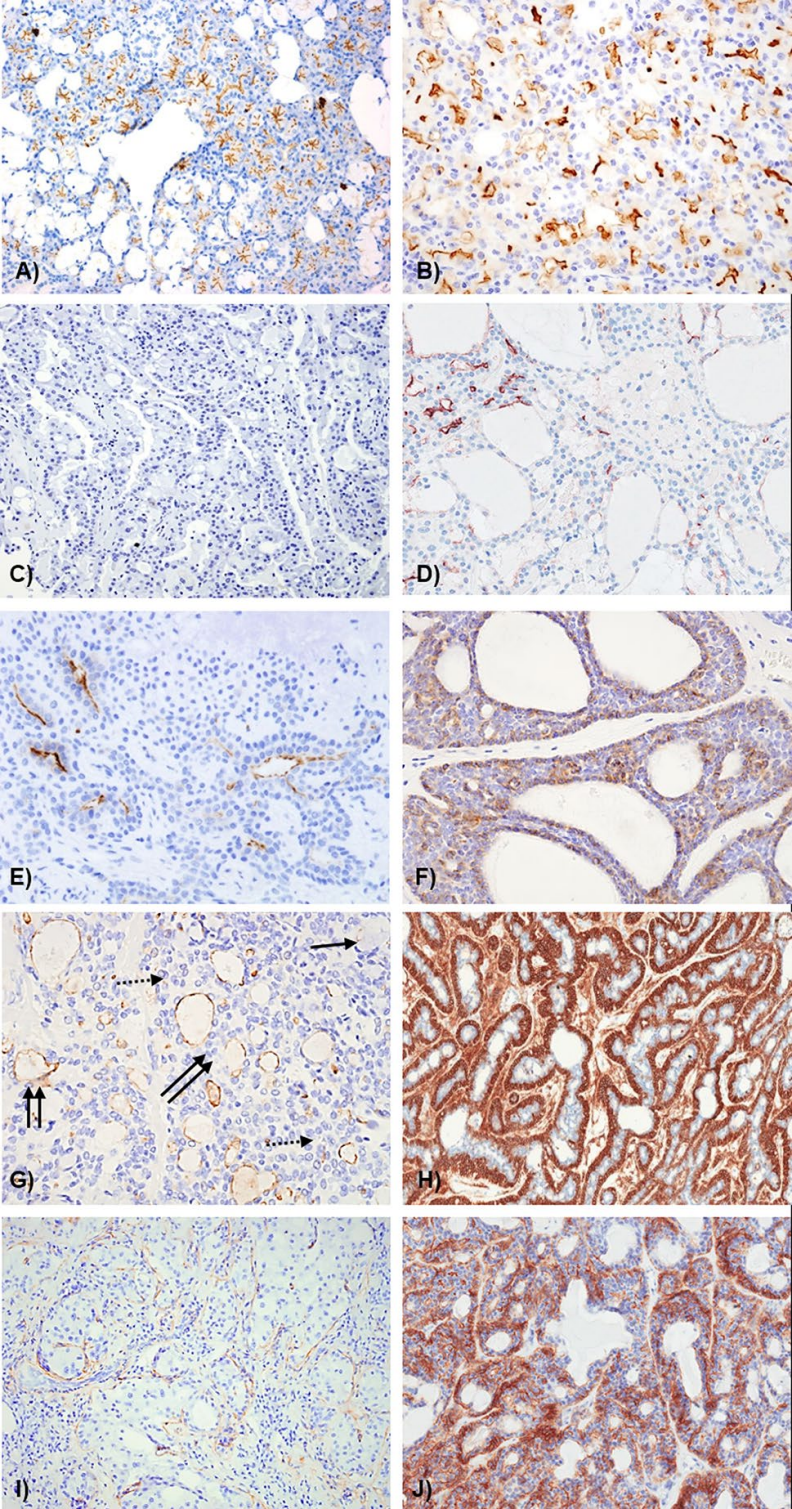
Representative photomicrographs showing DOG-1 staining pattern in **a** normal gland; **b** acinic cell CA; **c** secretory carcinoma—negative; **d** secretory carcinoma—focal positive, **e** pleomorphic adenoma; **f** adenoid cystic CA; **g** mucoepidermoid carcinoma; **h** basal cell adenoma; **i** carcinoma in pleomorphic adenoma; **j** papillary cystadenocarcinoma. **g** Solid arrow—mucous cells, dotted arrow—intermediate cells, double arrow—luminal spaces with DOG1 positivity

Strong apical/luminal DOG1 staining was seen in normal acini (10/10) (Fig. 1a), although occasional cells demonstrated lateral and basal expression. Staining was stronger in serous acini compared to mucous and focally intercalated ducts showed positive luminal reactivity. A similar staining pattern was seen in ACC, with widespread luminal DOG1 staining in 14 of the 15 cases (Fig. 1b). SC however were largely negative (Fig. 1c) with only weak focal luminal staining in three cases with a microcystic architecture (3/9) (Fig. 1d). SC with a papillary cystic architecture or clear cell change were negative. Variable luminal staining was seen in PA (14/49) (Fig. 1d), PAC (4/6—not shown), Ca ex-PA (4/11) (Fig. 1i), AdCC (6/20) (Fig. 1e). MEC showed luminal and/or mucous cell staining in 8/11 cases (Fig. 1f). BCA showed strong staining of myoepithelial cells in 5/14 cases (Fig. 1h), and some myoepithelial staining was also seen in PA, Ca ex PA, BCA, SDC, AdCC and PCAC (Fig. 1; Table 2). No staining was seen in myoepitheliomas (0/6), MC (0/2), CA (0/19), AdNOS (0/4) and BCAC (0/2).

**Table 2:** Summary of DOG1 staining in salivary gland neoplasms

Site	Cases	+ve	Pattern
Normal gland	10	10/10	Acini & ducts—luminal
Acinic cell carcinoma	15	14/15	Diffuse luminal in acini and small ducts
Secretory carcinoma	9	3/9	Negative or weak/focal luminal in microcystic areas
Pleomorphic adenoma	49	14/49	Focal luminal or myoepithelial (5/14)
Ca ex PA	11	4/11	Luminal and/or myoepithelial cells (2/4)
Myoepithelioma	6	0/6	N/A
Myoepithelial carcinoma	2	0/2	N/A
Adenoid cystic carcinoma	20	6/20	Weak abluminal + luminal
Polymorphous adenocarcinoma	6	4/6	Focal luminal
Basal cell adenoma	14	5/14	Luminal or abluminal/myoepithelial—variable (5/5)
Canalicular adenoma	19	0/19	N/A
Mucoepidermoid carcinoma	11	8/11	Luminal + mucous cell brush borders
Oncocytoma	2	0/2	N/A
Adenocarcinoma NOS	4	0/4	N/A
Basal cell adenocarcinoma	2	0/2	N/A
Salivary duct carcinoma	3	1/3	Myoepithelial cells (1/1)
Papillary cystadenocarcinoma	1	1/1	Abluminal/myoepithelial cells (1/1)
Total	184	70	

For each tumour type the total number of positive cases is given in relation to the total number of the cases

Myoepithelial DOG-1 staining was compared with a range of existing markers including αSMA, calponin, p63, S100 and CK14.

### α-Smooth Muscle Actin (αSMA)

Widespread αSMA staining was seen in myoepithelial cells surrounding the acini in normal salivary glands. The majority of the ACC and SC were negative for αSMA with focal staining seen in only 4/14 and 3/9 of cases. More consistent myoepithelial staining was seen in PA, Ca ex PA and myoepithelioma whereas PAC was negative.

Strong αSMA staining was also seen in abluminal cells of both tubular and cribriform AdCC (17/20 cases) consistent with myoepithelial cells. The three negative cases included two solid and one tubular AdCC.

Almost all cases of PAC were αSMA negative (not shown) whereas all basal cell adenomas demonstrated strong αSMA staining in the abluminal, myoepithelial cells. However, areas lacking **α**SMA expression were also observed.

### Calponin

**Fig. 2:**
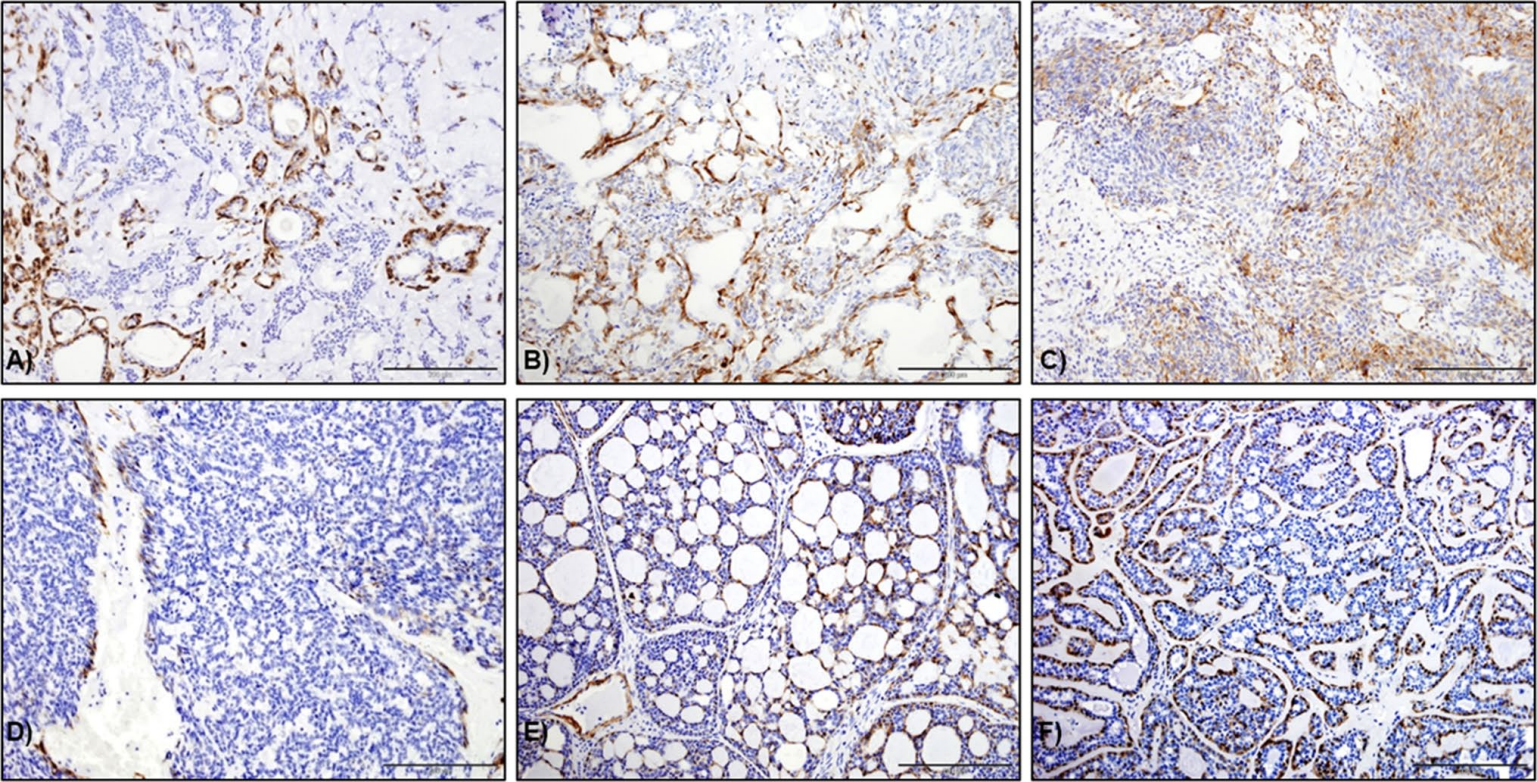
Calponin staining in **a** pleomorphic adenoma, **b** Ca ex-pleomorphic adenoma, **c** myoepithelioma and **d** PAC. Staining was predominantly seen in myoepithelial cells surrounding the ductal areas in (**a**, **b**). In **c**, scattered staining in spindle cells was seen throughout. PAC were negative except with one case showing focal staining. **d** Calponin staining highlighting the abluminal/myoepithelial cells Cribriform AdCC and **e** tubular AdCC (original magnification ×20)

ACCs and SC did not stain for calponin. Staining was seen in most cases of pleomorphic adenoma (44/47; 93.6%) (Fig. 2a). Staining was frequently observed in abluminal and spindled myoepithelial cells whereas plasmacytoid cells were always negative. Similar to PA, 90% (9/10) of Ca ex-PA showed Calponin staining in abluminal cells in addition to scattered stromal cells (Fig. 2b). Calponin staining was seen in myoepithelial cells in 66.7% of myoepitheliomas (4/6) (Fig. 2c). Focal staining was seen in myoepithelial carcinomas whereas PAC were largely negative with only one case showing some focal staining (Fig. 2d).

**Fig. 3:**
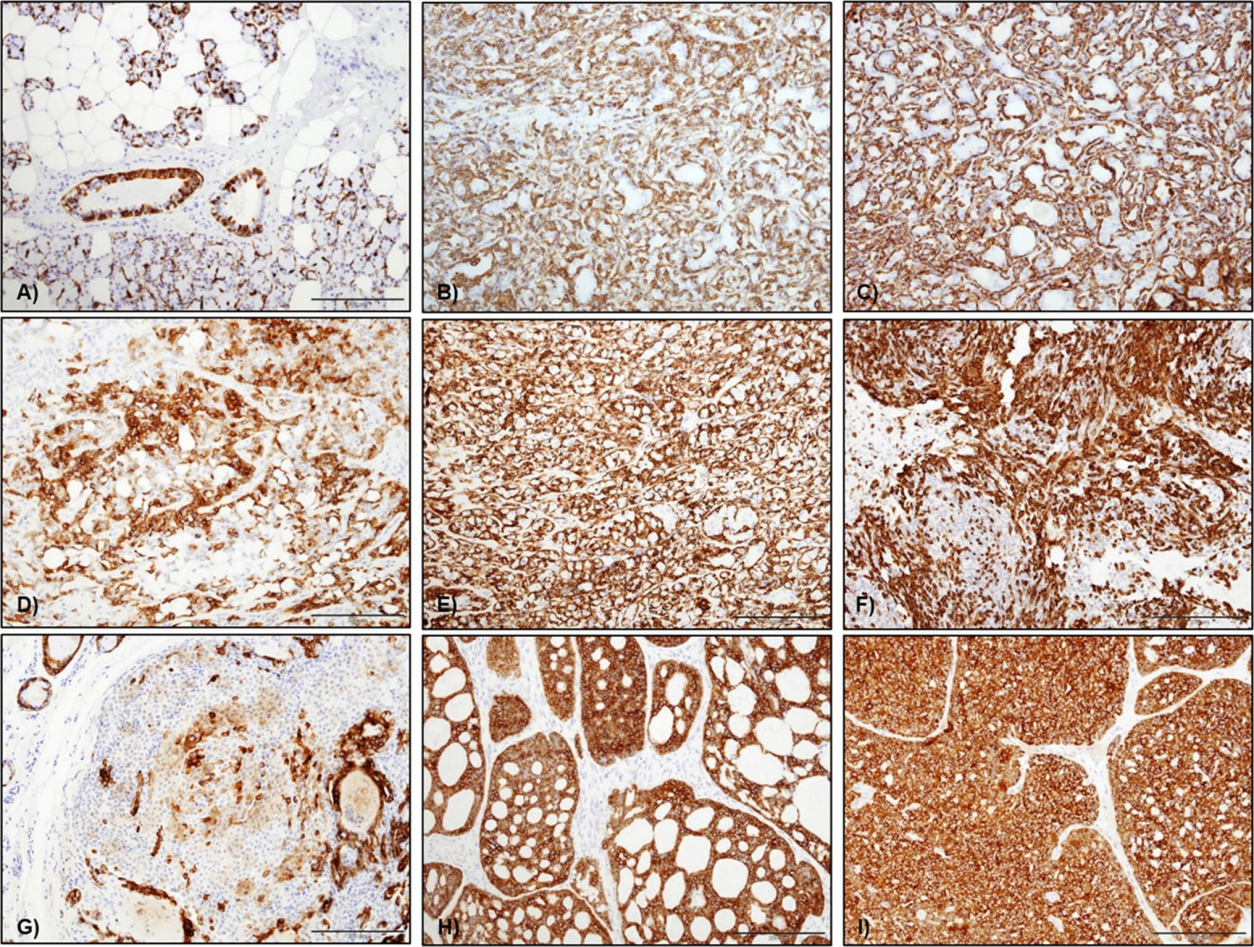
CK14 staining in salivary gland tumours **a** in normal tissue CK14 staining was mainly seen as a cytoplasmic staining of myoepithelial cells surrounding acini and in some basal cell in ducts, **b** CK14 in ACC was variable with one case showing diffuse abluminal staining, **c** CK14 in one SC with diffuse cytoplasmic staining of abluminal cells, **d** CK14 staining in PA was predominantly seen in the cytoplasm of most tumour cells, but plasmacytoid cells were negative, **e** CK14 expression in CA ex-PA was mainly seen in the cytoplasm of the abluminal type cells, **f** CK14 staining in myoepithelioma was variably positive in the cytoplasm of the neoplastic myoepithelial cells, mainly spindle cells, **g** CK14 staining in MC was focal with cytoplasmic staining of scattered tumour cells, **i** CK14 staining in AdCC (cribriform variant) and **j** PAC—diffuse staining was seen throughout the tumour (original magnification ×20)

Calponin staining was seen in 16/20 (80%) AdCC with expression in myoepithelial cells in both cribriform (Fig. 2e) and tubular (Fig. 3f) variants whereas the three negative cases had solid and mixed patterns.

### CK14

In normal salivary glands, CK 14 staining was seen in myoepithelial cells surrounding acini and ducts (Fig. 3a). Both ACC and SC were largely negative for CK14; however 4/16 ACC showed focal CK14 staining (Fig. 3b) with diffuse staining seen in one SC (Fig. 3c).

For PA, 89.6% (43/48) of the cases were CK14 positive with staining of both ductal and myoepithelial cells. However, staining was weak or absent in plasmacytoid cells, and cells in myxochondroid areas (Fig. 3d). CK 14 was diffusely positive in 90.9% of Ca-ex-PA (10/11) with strong staining in myoepithelial and abluminal cells throughout the tumours (Fig. 3e).

In myoepithelioma, CK14 was variably positive in myoepithelial cells in 5/6 of the cases (83.3%), mainly in cells with spindled morphology (Fig. 3f). Both cases of myoepithelial carcinoma showed focal staining of the tumour cells (Fig. 3g).

CK 14 staining in AdCC was observed in 13 out of 14 examined cases with strong diffuse staining in both luminal and abluminal cells (Fig. 3h). Variable reactivity of the tumour cells was seen in the tubular variant which also showed weaker staining intensity compared to tumours with a cribriform pattern. The only negative AdCC case had a solid architecture.

All cases of PAC showed diffuse CK14 staining. The staining was strong and diffuse throughout tumour cells (Fig. 3i). The variable expression profile of CK14 between different tumours indicates that it is not a reliable or specific myoepithelial marker.

### S100

Diffuse S100 staining was seen in myoepithelial cells in normal glands. Some ACC showed weak focal staining in acini, however most were negative. Cases with solid and papillary cystic patterns were completely negative for S100.

**Fig. 4:**
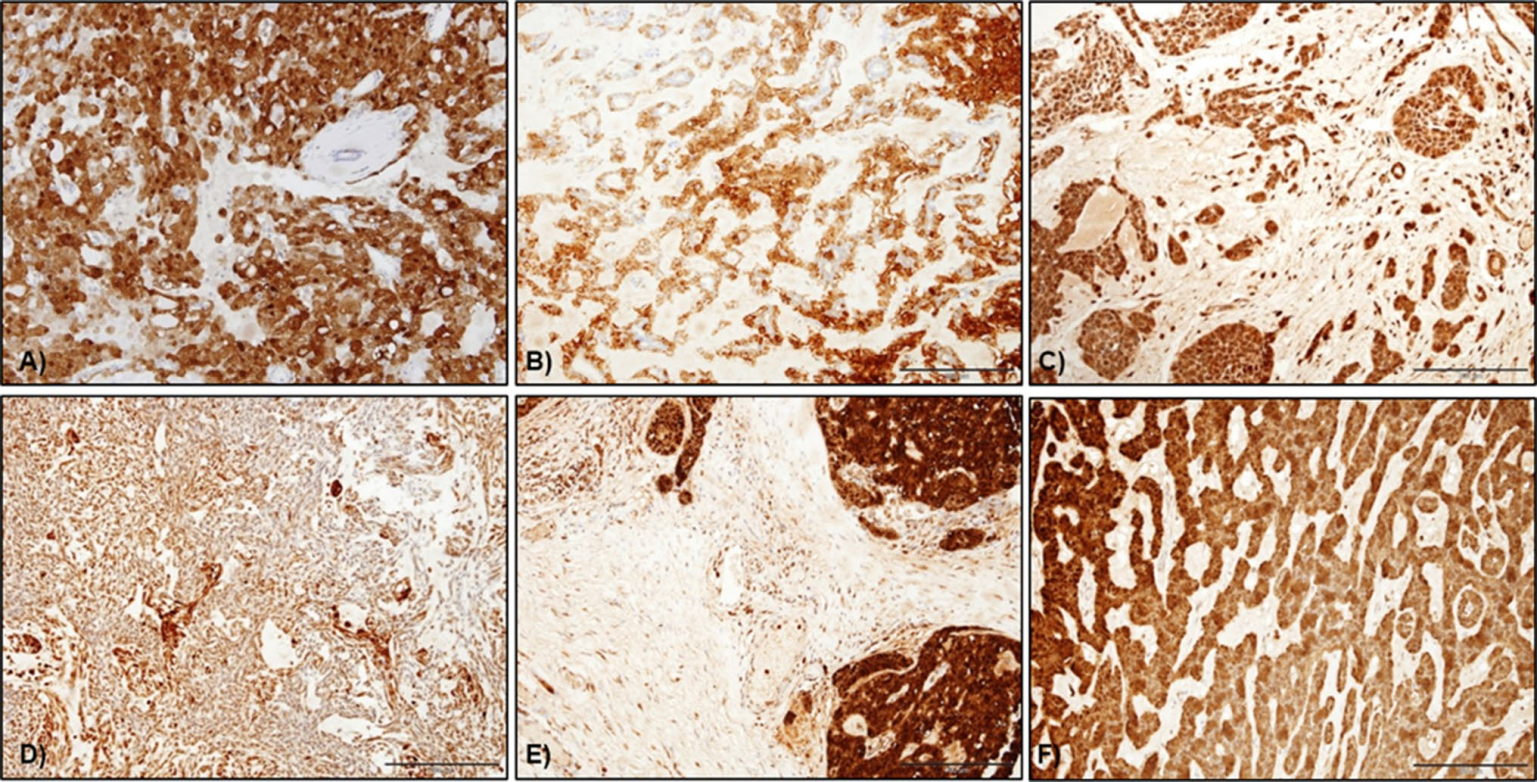
S100 staining in salivary gland tumours. **a** Nuclear and cytoplasmic staining in myoepithelial cells in PA whereas luminal cells were largely negative, **b** S100 expression in Ca ex-PA with diffuse staining in the cytoplasm of luminal, abluminal and scattered stromal cells, **c** myoepithelioma showing diffuse cytoplasmic S100 staining in the neoplastic spindle cells, **d** S100 staining in MC with strong cytoplasmic staining in the plasmacytoid cells, **e** PAC showed diffuse staining in almost all the tumour cells (original magnification ×20)

All cases of SC (9/9) were S100 positive and showed strong and diffuse staining of nuclei and cytoplasm of tumour cells (Fig. 4a). All PA (44/44; 100%) showed diffuse S100 staining including in spindle and plasmacytoid myoepithelial cells as well as cells within myxoid tissue (Fig. 4b). All cases of Ca-ex-PA (11/11) showed similar reactivity (Fig. 4c).

There was strong and diffuse S100 reactivity in all myoepitheliomas (6/6) and myoepithelial carcinomas (2/2) including spindled and plasmacytoid cells (Fig. 4d, e). All examined cases of AdCC (14/14) were positive for S100. Staining was observed mainly in the luminal cells of the ducts or cystic structures in the tubular and cribriform cases with some showing both luminal and abluminal staining. Diffuse staining was seen in the solid variant, but with less intensity. All five cases of PAC (100%) were positive for S100, with all the neoplastic cells showing diffuse strong to moderate cytoplasmic staining (Fig. 4f).

### p63

In ACC, 14/16 (87.5%) of the examined cases were p63 negative, with only two cases showing focal staining. Staining in SC was also variable with only 3/9 cases showing p63 positivity (not shown).

44/49 cases of PA (89.8%) showed p63 staining in abluminal and myoepithelial cells. Ca-ex-pleomorphic adenoma showed a similar staining distribution, pattern and intensity in 90.9% (10/11) of the cases. 100% of myoepitheliomas (6/6) showed strong nuclear staining in spindle and plasmacytoid cells. However, only one case of myoepithelial carcinoma (1/2) was positive, with focal areas of nuclear staining of the tumour cells.

In AdCC, 9/12 (75%) of the cases were p63 positive mainly in the abluminal cells, whereas luminal cells in ductal and tubular areas were negative. The negative cases showed a mostly solid pattern. All cases of PAC and BCA showed p63 staining in abluminal cells with stronger and more widespread staining seen in BCA.

Comparison with other myoepithelial markers showed that all cases with DOG-1 staining in myoepithelial cells were also positive for αSMA, calponin, CK14 and p63. In addition, only a limited number of PA, Ca ex PA and AdCC showed abluminal and/or myoepithelial DOG-1 positivity compared to other markers.

## Discussion

### DOG-1 Expression

DOG1 has been reported as a marker for differentiated acinic cells and intercalated duct cells [6], and is thought to be particularly useful for the diagnosis of ACC. In the current study, we examined a wide range of salivary neoplasms to determine the expression profile and distribution of DOG1 and report expression in myoepithelial cells for the first time.

ACC showed diffuse luminal DOG1 staining in 14/15 (93.3%) of the examined cases in agreement with the literature and similar to the staining pattern seen in the acini of normal glands [6, 7, 10, 11, 13–17]. Staining of duct cells was patchy and weak but widespread luminal staining was evident in all tumours. The majority of SC lacked DOG1 staining, similar to previous reports [6–8, 15, 18], confirming the diagnostic usefulness of DOG-1 in differentiating between ACC and SC.

28% of pleomorphic adenomas showed some DOG-1 staining with a predominantly apical/luminal pattern as previously reported [6, 17]. However, staining in myoepithelial cells was seen in 5 cases, which is a novel finding. A similar pattern was seen in carcinoma ex PA with apical/luminal staining in addition to myoepithelial staining in two cases whereas no staining was evident in myoepitheliomas or myoepithelial carcinomas.

Only two cases of AdCC showed weak focal reactivity for DOG-1. The first had a tubular pattern with DOG1 staining of the luminal aspect of the tubules, while the second showed a mixture of solid and cribriform patterns with occasional cells staining towards the periphery of the solid tumour islands. These are somewhat different to previous findings [6, 19] which reported consistent luminal staining within the cribriform areas. The reason for this difference is not entirely clear but lack of DOG1 staining in AdCC and difference between antibody clones has been reported by other groups [17].

Expression of DOG1 in PAC was only seen in two of five cases and showed only focal luminal expression similar to a previous report [6]. Somewhat similar to our findings, Montalli et al. reported cytoplasmic DOG1 staining in a 4/21 PACs in their cohort with cytoplasmic or occasionally apical staining [11].

Five cases of basal cell adenoma showed strong and diffuse staining for DOG1 in abluminal cells, which was similar to the pattern of αSMA and p63 staining, and was consistent with strong expression of DOG-1 on myoepithelial cells. Luminal staining was also seen focally in two cases. Montalli et al. suggested that staining was luminal in tubular and abluminal in non-tubular BCAs but we did not observe an obvious correlation between histological and staining patterns [20]. However, our BCA cohort was smaller including solid, tubular and non-tubular variants making it difficult to establish a relationship between morphology and staining.

MEC showed focal staining for DOG1 in 8 cases. In 3 this was weak luminal staining, and in the remainder there was weak or faint expression at the margins of mucous cells. This is somewhat similar to a previous study reporting weak staining in mucous and intermediate cells in MEC [19]. Canberk et al. studied FNAs from salivary tumours reporting DOG-1 cytoplasmic staining in 14% of cases but did not describe the precise location of the staining [17]. Myoepithelial staining for DOG1 was also seen in one adenocarcinoma NOS and a papillary cyst adenocarcinoma.

### Expression of Myoepithelial Markers

Calponin appeared to the most specific and sensitive myoepithelial marker followed by αSMA and p63. In some AdCC, PA and BCA, DOG-1 variable staining in myoepithelial cells was seen. ACC and SC were largely negative for myoepithelial markers similar to previous reports [7, 21–23]. S100 was very useful in differentiating between these two entities as strong diffuse S100 staining was seen in all SC but ACC were negative except for occasional focal and weak staining. This supports previous reports in the literature [7, 14, 24–27] and further confirms the diagnostic usefulness of S100 in SC [8].

αSMA was expressed in the majority of PA in abluminal and myoepithelial cells whereas plasmacytoid cells were negative [28, 29]. Calponin staining was present in all analysed cases including plasmacytoid cells indicating higher sensitivity than αSMA [30]. Abluminal/myoepithelial staining for S100 and p63 was also seen in all cases and similar profile was exhibited by Ca ex-PA [26, 31, 32]. αSMA, calponin and p63 staining in AdCC was variable, but was mainly seen in myoepithelial cells surrounding tubular and cribriform structures [33, 34]. Almost all cases demonstrated diffuse luminal and abluminal staining for CK14, suggesting a lack of specificity. In PAC, staining for αSMA and calponin were largely negative, but diffuse CK14 and S100 staining was seen in most tumour cells [35, 36] whereas p63 staining was limited to abluminal cells [37].

## Conclusion

Absence of luminal DOG-1 staining can differentiate ACC from SC, but variable staining is seen in PA, PLGA and Ca ex-PA. Myoepithelial staining in some tumours but not in normal gland is an interesting finding suggesting a wider distribution in SGT than originally envisaged. However, DOG-1 staining in myoepithelial cells appears inconsistent and not as reliable and sensitive as the existing markers, limiting its diagnostic utility.
